# Isolation, Molecular Characterization, and Biological Control of *Fusarium solani* Causing Stem Rot of Durian Tree in China

**DOI:** 10.3390/pathogens15080849

**Published:** 2026-08-14

**Authors:** Wenjing Jiang, Haoyu Wei, Hongwei Gao, Zihan Zhu, Qurban Ali, Xuewen Gao

**Affiliations:** 1 Sanya Institute of Nanjing Agricultural University, Sanya 572024, China; wenjingjiang@stu.njau.edu.cn (W.J.); haoyu@stu.njau.edu.cn (H.W.); gaohongwei@stu.njau.edu.cn (H.G.); zihanzhu@stu.njau.edu.cn (Z.Z.); 2 State Key Laboratory of Agricultural and Forestry Biosecurity, College of Plant Protection, Nanjing Agricultural University, Nanjing 211800, China; 3 Department of Biology, College of Science, United Arab Emirates University, Al-Ain 15551, United Arab Emirates; rattarqurban@uaeu.ac.ae

**Keywords:** durian stem rot, *Fusarium solani*, *Bacillus subtilis*, biological control, sustainable disease management

## Abstract

Durian (*Durio zibethinus* L.) is a high-value tropical fruit crop that has recently been cultivated on a commercial scale in Hainan Province, China. However, emerging diseases increasingly threaten sustainable production and fruit quality. Field surveys conducted in Sanya, Hainan, identified stem rot as the predominant disease affecting durian trees, with a disease incidence of approximately 26.6%. Affected trees exhibited necrotic lesions at stem-branch junctions, followed by leaf abscission and progressive decline. The causal agent was identified using morphological characteristics, phylogenetic analyses of the internal transcribed spacer (*ITS*) and translation elongation factor 1-alpha (*TEF-1α*) and RNA polymerase II second largest subunit (*RPB2*) gene regions, and pathogenicity assays. Inoculated plants developed symptoms consistent with those observed under field conditions, thereby fulfilling Koch’s postulates. Both morphological and multilocus molecular analyses consistently identified the pathogen as *Fusarium solani*. A total of 20 *Fusarium* isolates were obtained from diseased durian stem samples. To our knowledge, this is the first report of *F. solani* causing stem rot of durian in Hainan Province, China. In addition, *Bacillus subtilis* SYST2 exhibited strong antagonistic activity against *F. solani* isolates FS-1, FS-2, and FS-3 in vitro, with inhibition rates of 55.7%, 54.6%, and 53.3%, respectively. Field trials further demonstrated that application of SYST2 significantly suppressed disease development, achieving a biocontrol efficacy of 53.51%. These findings establish *F. solani* as a causal agent of durian stem rot in Hainan and highlight the potential of *Bacillus*-based biological control as a sustainable strategy for disease management in durian production systems.

## 1. Introduction

Durian (*Durio zibethinus* L.), which originated in Borneo, belongs to the genus *Durio* in the family Malvaceae [1]. It is one of the most economically important tropical fruit crops in Southeast Asia [2]. Due to its unique flavor, rich nutritional value, and high economic return, durian is widely known as the “king of fruits” [3]. Currently, Thailand is the world’s largest producer and exporter of durian, followed by Malaysia and Indonesia, while China has become one of the largest importers and consumers of the fruit [4]. To reduce dependence on imported durian and promote the development of the tropical agricultural industry, China began commercial durian cultivation in Hainan Province in 2019 and has since achieved substantial progress. By 2024, the cultivation area in Hainan had expanded to approximately 267 hm^2^, with an annual production of about 2000 tons. The widely cultivated varieties include ‘Monthong’, ‘Musang King’, ‘Kanyao’, and ‘Black Thorn’ [5,6]. However, as a typical tropical fruit tree, durian grows best under warm and humid conditions, which also favors the occurrence and spread of various plant pathogens [7]. As a result, plant diseases have increasingly emerged as a major challenge limiting the sustainable development of the durian industry [8].

Previous studies have shown that durians are susceptible to numerous diseases throughout their growth cycle, including root rot, stem rot, fruit rot, stem canker, leaf spot, leaf blight, and anthracnose [9,10,11,12,13]. Among these diseases, stem rot is considered one of the most destructive diseases affecting durian production and has been reported to occur severely in major durian-producing countries such as Thailand [10]. The disease mainly affects the trunk and bark, causing brown lesions that may expand into the vascular tissues, eventually resulting in wilting, chlorosis, and even plant death [14]. Several pathogens have been reported to be associated with durian stem rot, including *Fusarium solani*, *Lasiodiplodia pseudotheobromae*, and *Phytophthora* spp. [10,15]. In addition, stem canker, which exhibits symptoms like those of stem rot, typically develops near the basal stem and is considered a major durian disease in Malaysia. Species of *Phytophthora* spp. are recognized as the primary causal agents of stem canker [16]. Importantly, the pathogen composition associated with the same disease may differ considerably among geographical regions.

The biological characteristics of plant pathogens directly influence their pathogenicity, transmission efficiency, and the difficulty of disease management. Among them, *F. solani* is a soil-borne plant pathogenic fungus with a broad host range, capable of infecting a wide variety of economically important crops and causing various diseases, including fruit rot, stem rot, and root rot [17,18,19,20]. *F. solani* can produce a variety of biologically active secondary metabolites, among which fusaric acid is considered one of the important toxic metabolites involved in pathogenicity. Fusaric acid promotes pathogen infection by disrupting host cellular metabolism, inducing oxidative stress, and interfering with plant defense responses [21]. In addition, *F. solani* can survive for extended periods in soil through the formation of chlamydospores. Once established in agricultural fields, the pathogen is difficult to eradicate, thereby increasing the complexity and challenges associated with disease management [22]. These characteristics highlight the importance of early and accurate diagnosis of *F. solani*-associated diseases to facilitate timely implementation of effective management strategies.

At present, research on durian diseases in China remains at an early stage. Previous studies have reported that major diseases in Hainan, including root rot, fruit rot, and stem canker, are primarily caused by *Phytophthora palmivora* [15,23]. However, the pathogen composition associated with durian stem rot in Hainan has not been systematically investigated, and the etiology of the disease remains poorly understood. Therefore, a comprehensive approach integrating multiple identification methods is necessary to accurately determine the causal agents and provide a theoretical foundation for the development of effective disease management strategies [24,25]. Currently, management of durian diseases mainly relies on integrated approaches, including cultural practices, chemical control, and biological control [26]. Cultural practices, such as pruning infected tissues, can help reduce primary inoculum sources; however, these methods alone are often insufficient to effectively limit disease spread [27]. Chemical control can provide rapid suppression of disease development, but long-term and excessive application may result in pathogen resistance and concerns regarding pesticide residues [28]. For example, excessive use of fungicide has led to the emergence of resistant isolates of *P. palmivora* [29].

In contrast, biological control and plant growth-promoting rhizobacteria (PGPR) have gained increasing attention because of their environmental compatibility and potential for sustainable disease management [30,31]. Among these microorganisms, *Bacillus* spp. are particularly promising biocontrol agents (BCAs) because they can suppress phytopathogens through multiple mechanisms. These mechanisms include direct antagonism through the production of antimicrobial compounds, such as fengycin, surfactin, and bacillomycin [32,33], and volatile organic compounds (VOCs) [34,35]. In addition, *Bacillus* spp. may compete with pathogens for nutrients and colonization sites and can enhance plant resistance through the induction of host defense responses [31]. The combined action of these mechanisms may restrict pathogen growth and infection while improving plant tolerance to disease. Previous studies have demonstrated the potential of *Bacillus* spp. for the management of durian diseases, including reductions in durian stem canker severity of up to 62% [36]. Therefore, screening effective BCAs and evaluating their antagonistic and disease-suppressive effects are important steps toward developing sustainable alternatives or supplements to conventional chemical control.

Based on these considerations, we investigated the etiology of durian stem rot in Hainan, China, and explored biologically based approaches for its management. We integrated field surveys, pathogen isolation, morphological characterization, multilocus phylogenetic analyses of *ITS*, *TEF1-α,* and *RPB2*, and pathogenicity assays to identify the causal agent of durian stem rot and fulfil Koch’s postulates. We further evaluated the antagonistic strain *B. subtilis* SYST2 and the commercial microbial agent *B. subtilis* R31 for their ability to suppress the pathogen and reduce disease development. *Bacillus* spp. can antagonize fungal pathogens through multiple, potentially complementary mechanisms, including the production of antifungal metabolites and VOCs, competition for nutrients and colonization niches, and activation of host defense responses [32,33,34,35]. These mechanisms provide a biological rationale for assessing SYST2 and R31 as potential BCAs; however, the specific molecular mechanisms underlying their activity against the pathogen were not investigated in the present study. By integrating pathogen identification and pathogenicity with the evaluation of microbial antagonists, our study broadens the current understanding of durian stem rots from disease etiology towards biologically based disease management. To our knowledge, this study provides the first identification of the causal agent of durian stem rot in Hainan and establishes a framework for the development of sustainable management strategies for this emerging disease.

## 2. Materials and Methods

### 2.1. Field Survey and Sample Collection

In May 2025, a field survey was carried out at a durian plantation (Dazuiniao No. 1 Orchard, Youqi Agricultural Co., Ltd.) in Sanya, Hainan Province, China (18°29′40″ N, 109°19′51″ E). During the survey, typical symptoms were observed on the branches of durian trees, including grayish-white to light-brown lesions with rough surfaces, accompanied by exudation of yellow–brown, viscous gum. These lesions could expand into healthy tissues. Based on these characteristic symptoms, diseased tissue samples were collected for subsequent isolation and identification. To ensure the reliability and reproducibility of pathogen identification, sampling was conducted from May 2025 to March 2026. In total, eight sampling events were carried out. At each sampling site, six symptomatic samples were randomly collected. Prior to each sampling event, all sampling tools were disinfected with 75% ethanol. The collected samples were immediately placed in sterile, sealed bags, appropriately labeled, and temporarily stored in a portable insulated container with ice packs. All samples were transported to the laboratory under sterile conditions, and pathogen isolation was performed within 24 h of collection.

### 2.2. Isolation and Purification of the Pathogen

The pathogen was isolated using a conventional tissue isolation method [37]. Small stem segments (approximately 5 mm × 5 mm) were excised from the interface between healthy and diseased regions of durian plants. The samples were surface-sterilized with 1% sodium hypochlorite for 2 min, followed by 75% ethanol for 30 s, and then rinsed three times with double-distilled water (ddH_2_O). The sterilized tissues were dried on sterile filter paper, placed onto potato dextrose agar (PDA) plates, and incubated at 25 °C. One mycelial colony grown from the plant tissues’ hyphal tips were transferred to fresh PDA plates for purification. Pure cultures were subsequently obtained and used for further experiments.

Purified fungal isolates were cultured on PDA and incubated at 25 °C in the dark. Colony morphology and diameter were recorded after 5 d of incubation. Microscopic characteristics were examined by staining conidia with lactophenol cotton blue and observed under a Leica DM500 RH microscope (Leica Microsystems, Wetzlar, Germany) equipped with a 100× oil immersion objective lens [38]. Morphological features, particularly the presence of characteristic sickle-shaped macroconidia, were used for preliminary identification of the isolates. The PDA medium containing potato 200 g L^−1^ and glucose 20 g L^−1^ was prepared in our laboratory and used for fungal isolation and cultivation.

### 2.3. Molecular Identification and Phylogenetic Analysis

Genomic DNA was extracted from the fungal isolates using the cetyltrimethylammonium bromide (CTAB) method [39], and the extracted DNA was used as the template for subsequent PCR amplification. The *ITS*, *TEF1-α*, and *RPB2* genes were selected as molecular markers. Previous studies have demonstrated that multilocus phylogenetic analysis based on the combined *ITS*, *TEF1-α*, and *RPB2* gene sequences provides high resolution for distinguishing closely related species within the genus *Fusarium* [40]. The primers used are listed in Table S1. The PCR amplification program consisted of an initial denaturation at 95 °C for 5 min, followed by 35 cycles of denaturation at 95 °C for 90 s, annealing at the specific temperatures for each primer pair (Table S1) for 90 s, and extension at 72 °C for 1 min, with a final extension at 72 °C for 10 min [41,42,43]. PCR products were verified by 1% agarose gel electrophoresis and then sent to Tsingke Biotechnology Co., Ltd. (Sanya, Hainan, China) for sequencing. All primers used in this study were also synthesized by the same company. The obtained sequences were submitted to GenBank, and accession numbers were assigned. For molecular identification, sequences of the *ITS*, *TEF1-α*, and *RPB2* loci were initially queried against the NCBI nucleotide database using BLASTn (available online at: https://blast.ncbi.nlm.nih.gov/Blast.cgi, last accessed on 20 April 2026). Reference sequences representing closely related *Fusarium* taxa were subsequently retrieved from GenBank based on sequence similarity and taxonomic relevance. Sequences for each locus were aligned separately using ClustalW implemented in MEGA 7.0, inspected for alignment consistency, and then concatenated in the order *ITS–TEF1-α–RPB2* to generate a multilocus dataset. Phylogenetic relationships were inferred in MEGA 7.0 (The Pennsylvania State University, State College, PA, USA) using the Neighbor-Joining method with evolutionary distances calculated under the Kimura two-parameter model. Statistical support for individual nodes was evaluated using 1000 bootstrap replicates. *Macroconia sphaeriae* CBS 112770 was included as the outgroup to root the phylogeny. The resulting multilocus phylogeny was used together with morphological characteristics and sequence similarity to determine the taxonomic placement of the fungal isolates.

### 2.4. Pathogenicity Tests

A total of 20 *Fusarium* isolates were recovered and characterized based on colony morphology, pigmentation, and conidial features. The isolates were clustered into three reproducible morphological groups, with limited phenotypic variation within groups but clear differences among groups. One isolate displaying the characteristic phenotype of each group was therefore selected as a representative and designated FS-1, FS-2, and FS-3. Together, these isolates encompassed the morphological diversity observed across the isolate collection and were subsequently subjected to multilocus identification and pathogenicity assays. The remaining isolates were preserved at −80 °C in 20% glycerol. Three *Fusarium* isolates were selected for pathogenicity testing. One-year-old durian seedlings of the cultivars ‘Monthong’ and ‘Musang King’ were used as experimental materials. These seedlings were obtained from a commercial nursery in Sanya, Hainan Province, China. After transplantation into the greenhouse, all plants were uniformly sprayed with carbendazim to eliminate potential surface contaminants. To minimize any residual effects of the fungicide on pathogen infection, inoculation was carried out 15 d after treatment.

The *Fusarium* isolates were cultured in PDB at 25 °C while shaking at 120 rpm for 7 d. The culture was then filtered through three layers of sterile gauze to collect conidia for inoculation. The conidial suspension was washed once with ddH_2_O. The conidial suspension was determined using a hemocytometer (Erma, Saitama, Japan) and adjusted to 1 × 10^6^ conidia/mL. Pathogenicity was assessed using a wound inoculation method. A sterile needle was used to create a wound approximately 2–3 mm deep at the branching point of the durian stem. Subsequently, 10 μL of the conidial suspension was applied to the wound site, which was then sealed with Parafilm to prevent moisture loss. Each treatment consisted of six replicates, with sterile water used as the control. After inoculation, the plants were covered with black plastic bags for 24 h to maintain high humidity and promote infection. The coverings were then removed, and the plants were maintained in a greenhouse at 28 °C and 75% relative humidity.

Disease development was assessed after 21 d post-inoculation (dpi). Disease incidence was calculated as the percentage of infected inoculation sites relative to the total number of inoculated sites. Pathogenicity of each isolate was evaluated by measuring lesion size [44]. In addition, differences in host responses between the two durian cultivars were compared to assessing their relative susceptibility to the pathogen.

### 2.5. Antagonistic Activity of B. subtilis SYST2 Against Fusarium

A *B. subtilis* SYST2 preserved in this laboratory has been reported to have good plant growth-promoting and induced systemic resistance abilities, so this study selected this strain for research related to biological control [45]. Its antagonism against *F. solani* FS-1, FS-2, and FS-3 were evaluated using a dual culture assay. The *F. solani* isolates were first pre-cultured on PDA plates. Once actively growing, mycelial plugs (5 mm in diameter) were cut using a sterile cork borer and placed at the center of fresh PDA plates. A defined volume of the bacterial suspension (1 × 10^8^ CFU/mL) was then inoculated approximately 2.5 cm away from the fungal plug. LB medium (tryptone 10 g L^−1^, yeast extract 5 g L^−1^, and NaCl 10 g L^−1^) was used as a negative control. Each treatment was performed with three replicates. All plates were sealed with parafilm and incubated at 25 °C for 2–3 d, after which the antagonistic effects were observed and recorded. The colony radius of the pathogen was measured in both control and treatment groups, and the percentage inhibition of radial growth (PIRG) was calculated.

The PIRG was calculated using the following formula [46]:

PIRG (%) = [(R1 − R2)/R1 × 100]

R1 = Radius of the fungal colony on the control plate
R2 = Radius of the fungal colony on the test plate

### 2.6. Field Evaluation of the Biocontrol Efficacy of Bacillus spp. Against Durian Stem Rot

A field experiment was conducted at a durian plantation of Youqi Agricultural Co., Ltd. in Sanya, Hainan Province, China, to evaluate the effectiveness of different treatments for controlling durian stem rot. The study was arranged in a randomized complete block design (RCBD). The materials tested included a commercial chemical fungicide (propamocarb hydrochloride), a commercial microbial agent (*B. subtilis* R31), and *B. subtilis* SYST2 isolated in our laboratory. Before the application, both bacterial suspensions were adjusted to the same concentration (1 × 10^8^ CFU/mL), while the chemical fungicide propamocarb hydrochloride was diluted 100-fold according to the manufacturer’s recommendation.

Four treatments were established: T1, water control; T2, propamocarb hydrochloride; T3, R31; and T4, SYST2. Each treatment included three replicates (blocks), with three durian trees per replicate. All treatments were applied to the stems of trees showing typical symptoms of stem rot. Applications were performed at 7 d intervals, for a total of five times (at 0, 7, 14, 21, and 28 d). Disease assessments were conducted before the first application (d 0) and subsequently at 7, 14, 21, and 28 d after treatment initiation. The lesion area was measured and subjected to statistical analysis for 28 d. During the experimental period, the temperature and relative humidity were recorded by an automatic weather station installed in the plantation, which was developed and deployed by Jike Soft Information Technology Co., Ltd. (Changchun, Jilin, China). The mean temperature was 27.6 °C (range, 26.4–28.2 °C), and the average relative humidity was 64.4% (range, 60–68%).

Lesions were photographed at a fixed distance of 30 cm using a Canon EOS R50 digital camera (Canon Inc., Tokyo, Japan), with a reference scale placed adjacent to the lesions for calibration. Lesions were defined as distinct brown to dark brown discoloration on the stem surface, forming visually distinguishable boundaries from the surrounding healthy tissues. Image analysis was conducted using ImageJ version 1.53 (National Institutes of Health, Bethesda, MD, USA) [47]. The brown, discolored lesion area was manually outlined using the Freehand Selection tool, and its lesion size was quantified using the “Measure” function. Disease progression was evaluated based on the increase in lesion area, defined as the expansion relative to the initial measurement. Control efficacy was calculated using the following formula:

Control efficacy (%) = [(ΔA_control − ΔA_treatment)/ΔA_control] × 100

where ΔA represents the increase in lesion area over the observation period.

### 2.7. Data Processing and Statistical Analysis

Experimental data are presented as mean ± standard error (SE). Statistical analyses were conducted using GraphPad Prism 8 (San Diego, CA, USA). Before one-way analysis of variance (ANOVA), the assumptions of normality and homogeneity of variances were assessed using the Shapiro–Wilk and Bartlett’s tests, respectively. One-way ANOVA was then conducted, followed by Tukey’s multiple comparison test for post hoc analysis. For each ANOVA, the F-value and exact *p*-value were reported in the Results Section. Differences among treatments were considered statistically significant at *p* < 0.05. Significant differences are indicated by different lowercase letters.

## 3. Results

### 3.1. Symptoms of Durian Stem Rot Disease

**Figure 1:**
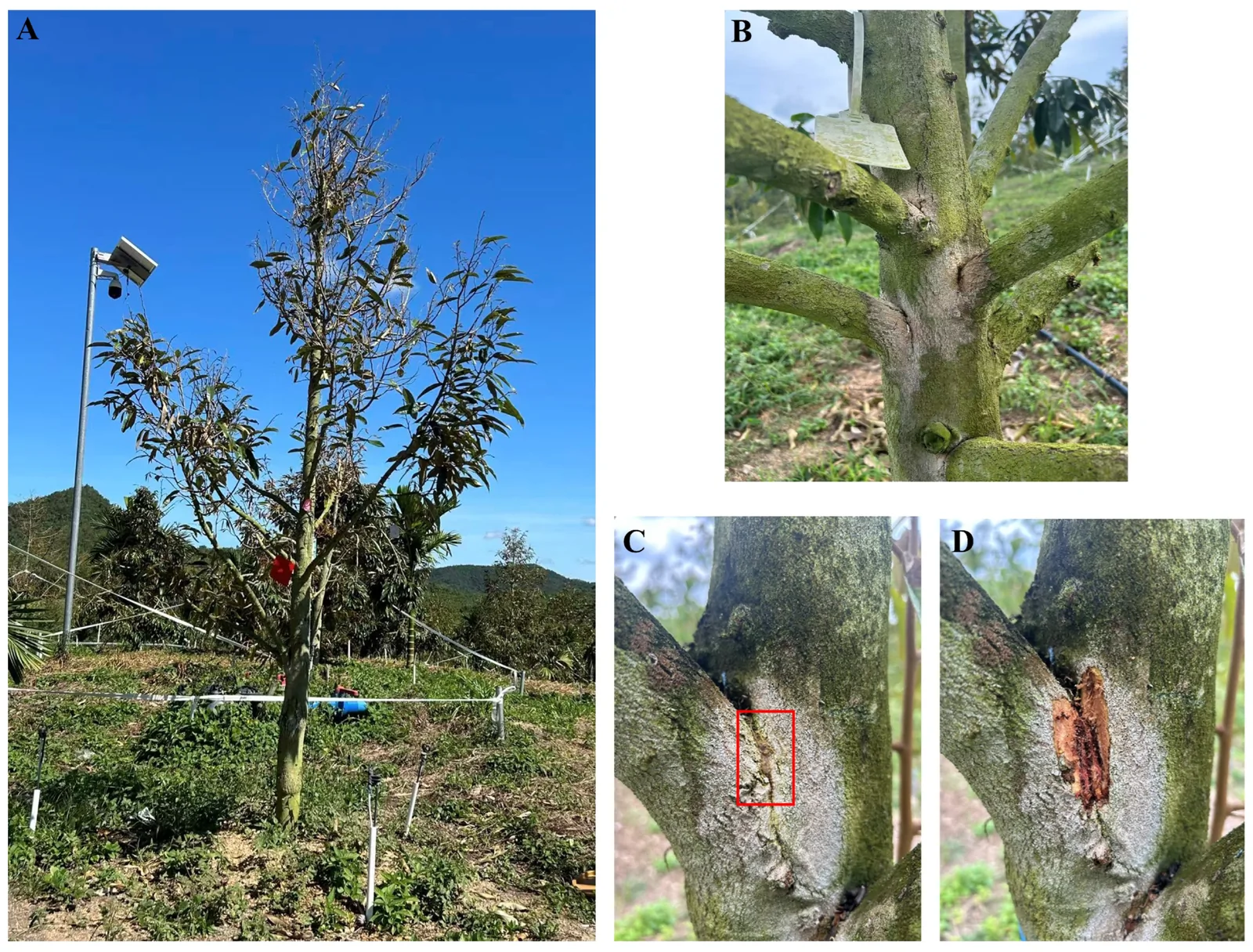
Symptoms of stem rot disease of durian in a plantation. (**A**) Aboveground symptoms of infected durian trees; (**B**) stem rot lesions on the trunk; (**C**,**D**) longitudinal sections of diseased tissues showing brown to black discoloration of vascular tissues.

In May 2025, a field survey of durian plantations was conducted in Sanya City, Hainan Province. Within an area of about 3 acres, 30 durian trees were examined, and 8 of them showed symptoms of stem rot disease, giving a disease incidence of 26.6%. The affected trees generally appeared weak, with sparse foliage and significant leaf drop (Figure 1A). Closer observations revealed that the disease mainly developed at the branch junctions of the trunk, where large lesions formed. These lesions had rough surfaces and varied in color from gray–white to light brown. Some trees had only a single lesion, while others showed three or more lesions (Figure 1B). In some infected trees, yellow–brown, sticky exudates were seen oozing from cracks at the center of the lesions (Figure 1C). The edges of the lesions were not clearly defined and gradually spread into the surrounding healthy tissue. When the diseased bark was cut longitudinally, brown to black necrotic discoloration was observed in the phloem tissues (Figure 1D).

### 3.2. Morphological Characterization

**Figure 2:**
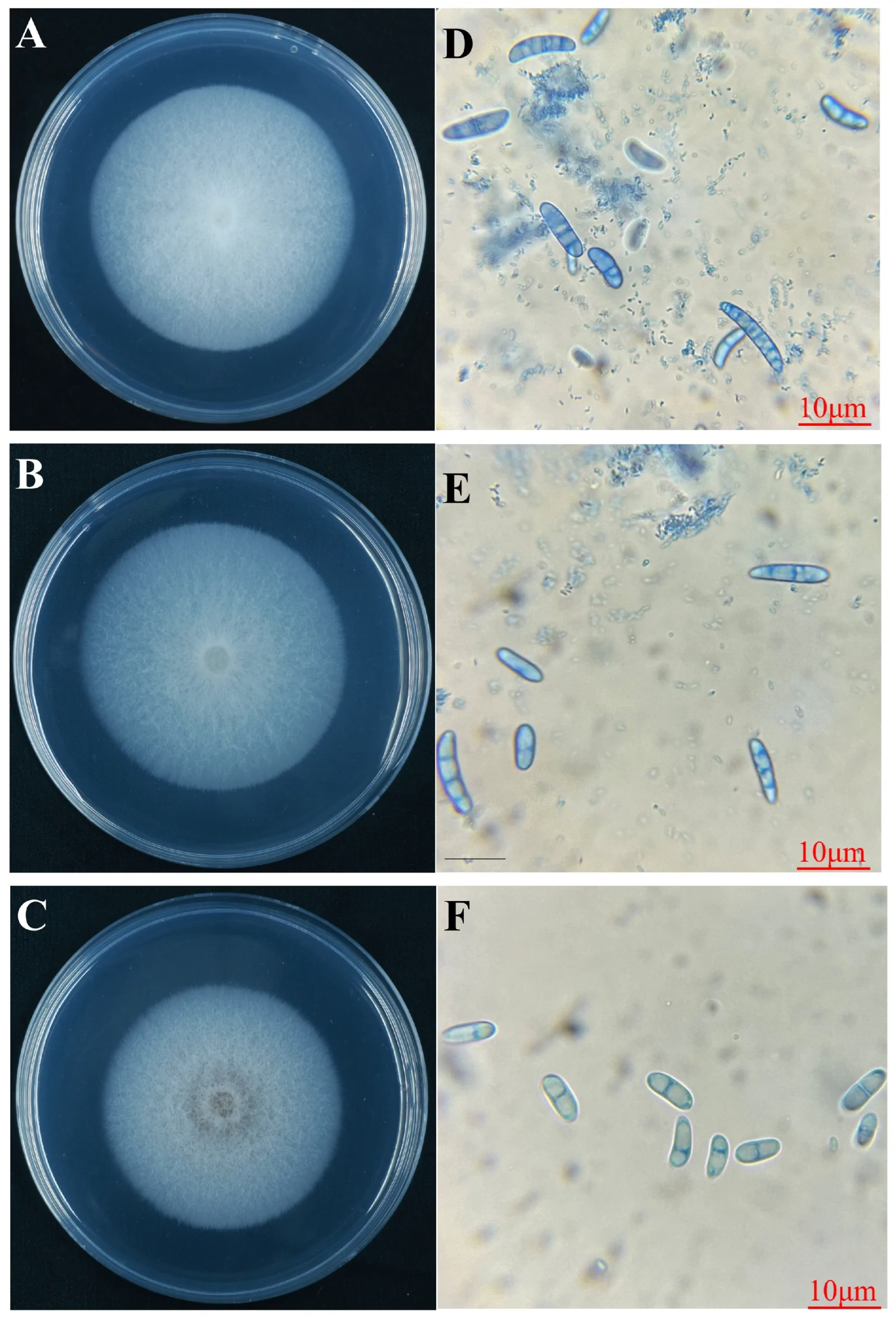
Morphological characteristics of the isolated strains. (**A**–**C**) Colony morphology of *F. solani* FS-1, FS-2, and FS-3, respectively. (**D**–**F**) Microscopic morphology of conidia observed under a Leica microscope with a 100× oil immersion objective. Scale bars = 10 μm.

From May 2025 to March 2026, eight rounds of fungal isolation were performed using diseased stem tissues collected from infected durian trees in the survey area to ensure the stability and reproducibility of the results. Multiple fungal isolates with similar morphological features were obtained, and all isolates had highly consistent colony morphology and growth characteristics. Based on differences in colony morphology, colony color, and conidial characteristics, three representative isolates (FS-1, FS-2, and FS-3) were selected for detailed characterization. After incubation on PDA medium for 5 d, the colony of FS-1 exhibited circular expansion, forming white, floccosum colonies with relatively dense aerial mycelia (Figure 2A). FS-2 showed similar growth characteristics to FS-1, with white colonies and more prominent but relatively loose aerial mycelia (Figure 2B). FS-3 initially produced white colonies, while light yellow to light brown pigmentation appeared in the central region at later stages. The aerial mycelia were relatively dense, and the colony margins were regular (Figure 2C). Microscopic observation revealed that FS-1 produced typical *Fusarium*-like conidia. The macroconidia were mainly spindle-shaped to falcate, slightly curved, gradually tapering toward both ends, and contained 2–3 septa; a small proportion of conidia exhibited up to six septa (Figure 2D). The macroconidia produced by FS-2 were mainly falcate in shape and typically contained 2–3 septa (Figure 2E). The microconidia produced by FS-3 were oval-shaped and mainly contained a single septum (Figure 2F). These morphological characteristics were consistent with those described by members of the genus *Fusarium* [48]. Therefore, isolates FS-1, FS-2, and FS-3 were preliminarily identified as *F. solani*.

### 3.3. Molecular Phylogenetic Analysis

**Figure 3:**
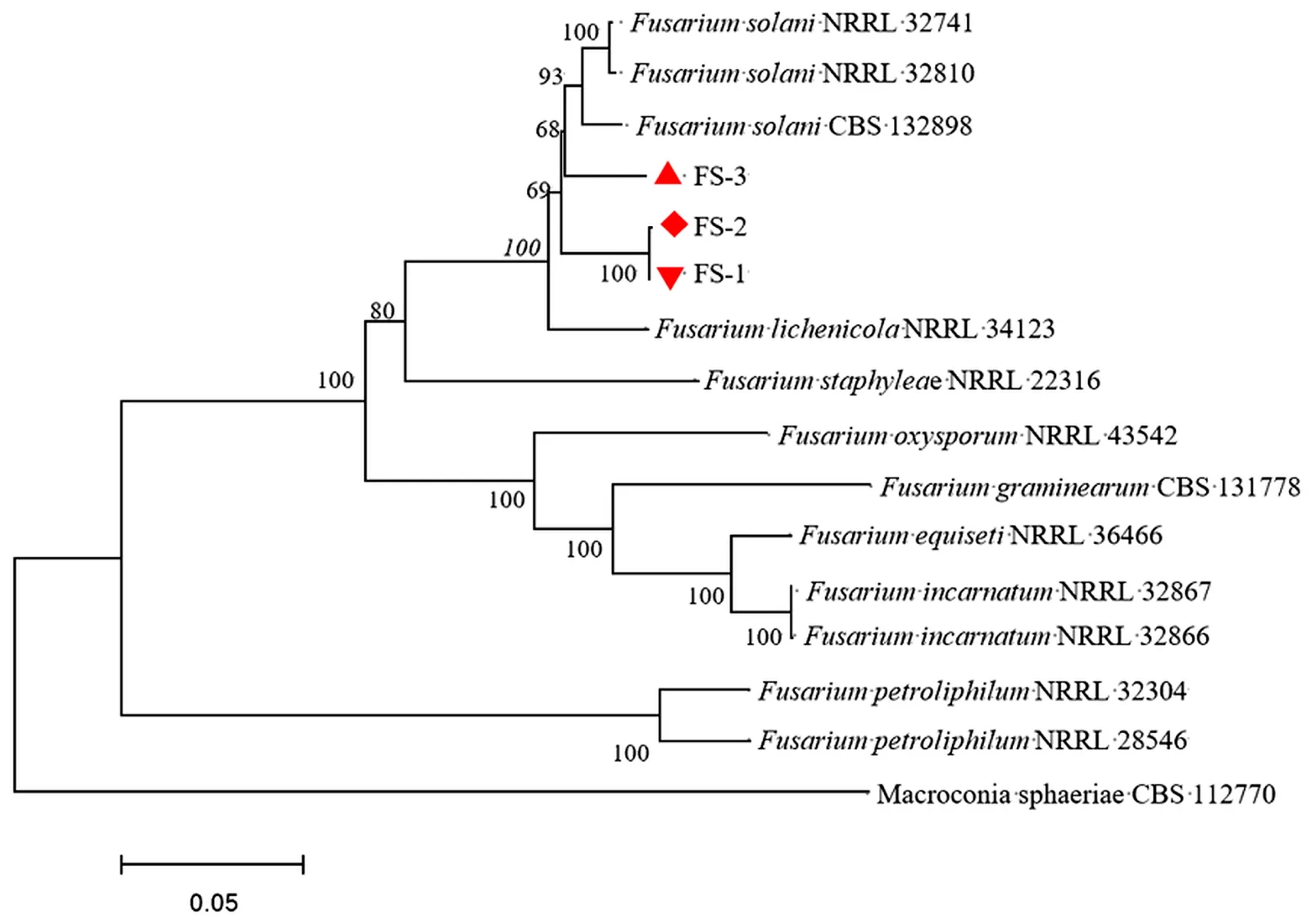
Phylogenetic tree of *Fusarium* isolates based on concatenated *ITS*, *TEF-1α*, and *RPB2* sequences constructed using the Neighbor-Joining (NJ) method. Evolutionary distances were computed using the Kimura 2-parameter (K2P) model. Bootstrap analysis was performed with 1000 replicates, and bootstrap values are shown at the branch nodes. Multiple sequence alignment was conducted using ClustalW implemented in MEGA 7.0. *M. sphaeriae* CBS 112770 was used as the outgroup to root the phylogenetic tree. The isolates obtained in this study are indicated with different symbols in the figure.

The *ITS*, *TEF1-α*, and *RPB2* gene sequences of strains FS-1, FS-2, and FS-3 were deposited in GenBank under the following accession numbers: FS-1: *ITS* (PZ312000), *TEF1-α* (PZ334101), *RPB2* (PZ802956); FS-2: *ITS* (PZ312001), *TEF1-α* (PZ334102), *RPB2* (PZ802957); FS-3: *ITS* (PZ312002), *TEF1-α* (PZ334103), *RPB2* (PZ802958). To further determine the taxonomic identity of the isolates, phylogenetic analysis was performed using reference *Fusarium* sequences retrieved from the NCBI database. A combined dataset of *ITS*, *TEF1-α*, and *RPB2* sequences was used for phylogenetic reconstruction, with *M. sphaeriae* CBS 112770 selected as the outgroup. The phylogenetic analysis showed that isolates FS-1, FS-2, and FS-3 are clustered within the *F. solani* species complex (FSSC) and grouped closely with reference strains NRRL 32741, NRRL 32810, and CBS 132898. In the phylogenetic tree, FS-1 and FS-2 first formed a well-supported monophyletic clade (Bootstrap = 100%), indicating a close phylogenetic relationship between them. Subsequently, FS-3 clustered with this clade and formed a secondary subclade (Bootstrap = 69%). However, FS-3 showed a certain phylogenetic distance from FS-1 and FS-2 (Figure 3). Overall, all three isolates were consistently positioned within the *F. solani* lineage. Combined with morphological characteristics, isolates FS-1, FS-2, and FS-3 were identified as *F. solani*.

### 3.4. Pathogenicity Test on Durian Seedlings

**Figure 4:**
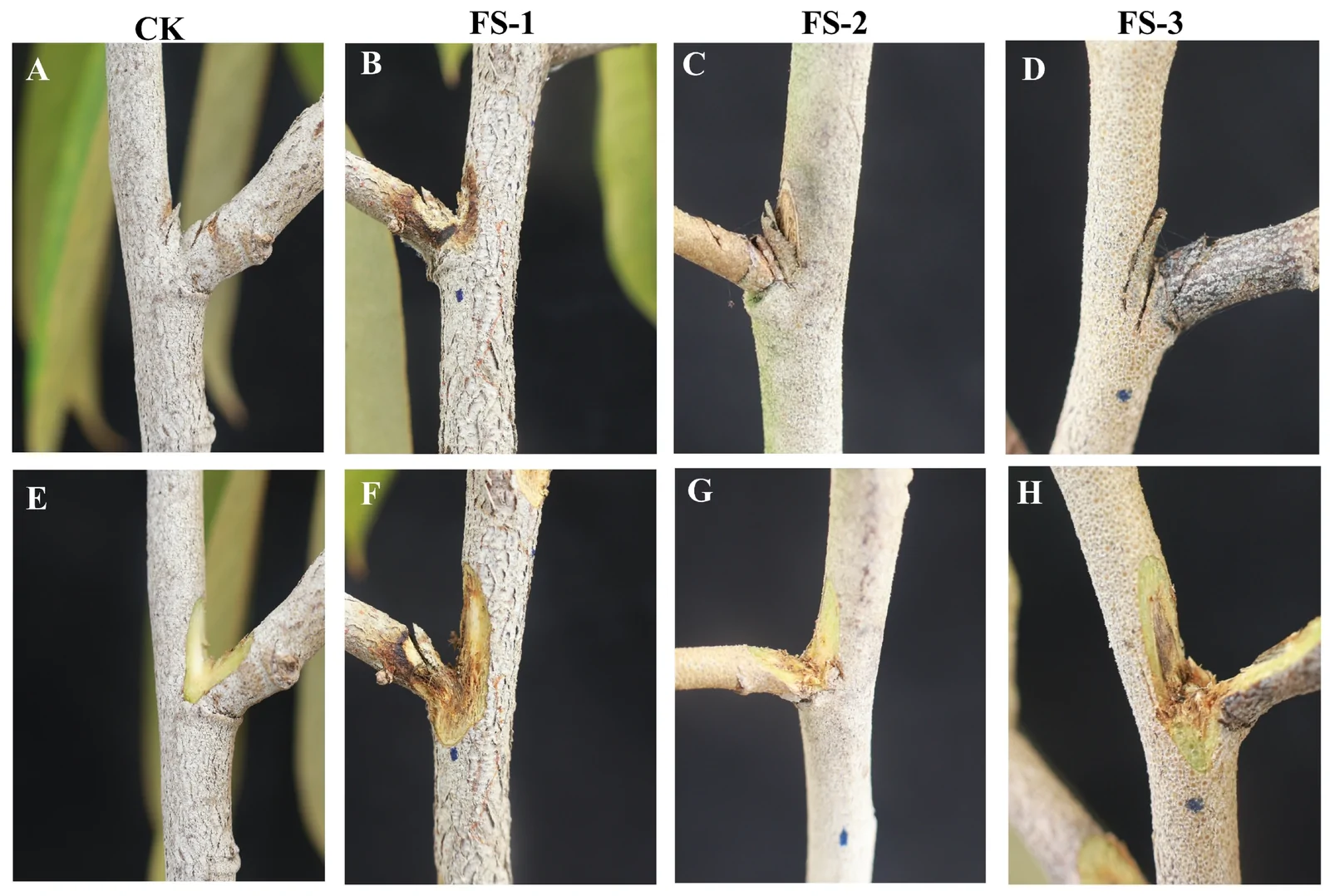
Pathogenicity of representative *Fusarium* isolates on durian seedlings. (**A**–**D**) Stem symptoms at 21 days post-inoculation and (**E**–**H**) corresponding longitudinal sections of infected tissues. (**A**,**E**) control; (**B**,**F**) FS-1; (**C**,**G**) FS-2; and (**D**,**H**) FS-3. The photograph was taken 21 days after inoculation.

Pathogenicity was performed on seedlings of two durian cultivars, Monthong and Musang King, using three *F. solani* isolates (FS-1, FS-2, and FS-3) through stem inoculation. The two cultivars exhibited markedly different responses to infection. No obvious disease symptoms were observed on Musang King seedlings throughout the experiment period, whereas typical stem rot symptoms developed on inoculated Monthong seedlings. After inoculation, lesions first appeared at the inoculation sites as grayish-white to light rough brown spots, while gradually expanding into the surrounding healthy tissues (Figure 4A–D). Longitudinal sections of infected stems revealed clear browning and necrosis of the phloem tissues (Figure 4E–H), consistent with symptoms observed under natural field conditions. No visible symptoms were detected in the control plants.

**Figure 5:**
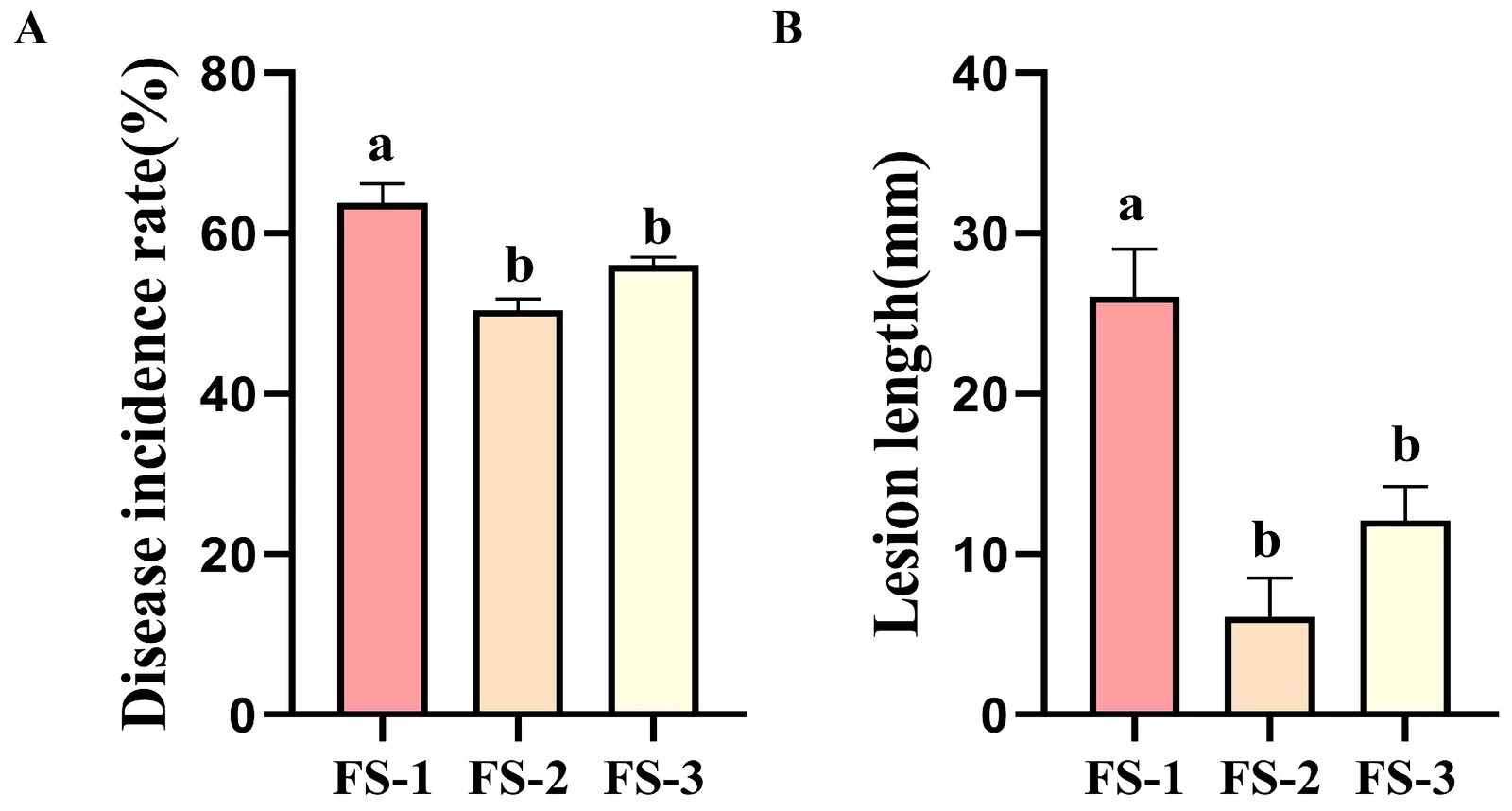
Pathogenicity evaluation of different isolates on durian seedlings. (**A**) Disease incidence after inoculation with *F. solani* isolates FS-1, FS-2, and FS-3; (**B**) lesion length at 21 days post-inoculation. Data are presented as mean ± SD. Different lowercase letters indicate significant differences among treatments (one-way ANOVA followed by Tukey’s multiple comparison test, *p* < 0.05).

The three isolates showed clear differences in pathogenicity. Disease incidence and lesion length were used to evaluate virulence. The disease incidence of ‘Musang King’ seedlings inoculated with FS-1, FS-2, and FS-3 was 0%, indicating that no disease symptoms were observed in any of the treated seedlings. For the inoculated ‘Monthong’ seedlings, disease incidence reached 61.22%, 49.33%, and 55.00%, respectively (Figure 5A). Lesion length was measured at 21 dpi. Lesion length differed significantly among treatments (F = 49.24, *p* = 0.0002), with FS-1 producing the largest lesions (25.24 ± 2.98 mm), compared with FS-2 (6.13 ± 2.40 mm) and FS-3 (12.10 ± 2.12 mm) (Figure 5B). These results demonstrate that FS-1 was the most aggressive isolate among the three tested isolates.

The pathogen was successfully re-isolated from infected tissues, and its identity was confirmed through both morphological characterization and molecular sequence analysis. The re-isolated strains were consistent with the original inoculated isolates, thereby fulfilling Koch’s postulates. These findings confirm that *F. solani* is the causal agent of durian stem rot. Overall, isolate FS-1 exhibited the highest pathogenicity, while ‘Monthong’ was more susceptible to infection than ‘Musang King’.

### 3.5. Antagonistic Activity of B. subtilis SYST2 Against F. solani

**Figure 6:**
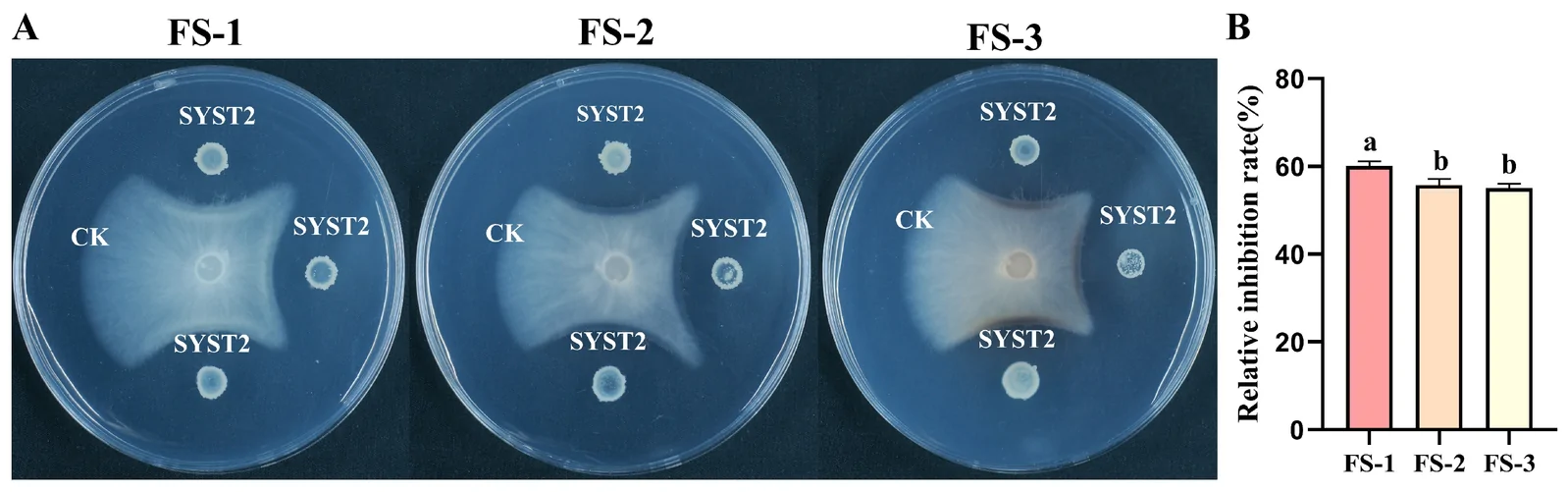
Antagonistic activity of *B. subtilis* SYST2 against *F. solani* FS-1, FS-2, and FS-3. (**A**) Dual culture assay on PDA plates; (**B**) relative inhibition rate. Data are presented as mean ± SD. Different lowercase letters indicate significant differences among treatments (one-way ANOVA followed by Tukey’s test, *p* < 0.05).

In vitro antagonistic assays demonstrated that SYST2 exhibited strong inhibitory activity against the three *F. solani* isolates FS-1, FS-2, and FS-3 (Figure 6A). Inhibition differed significantly among isolates (F=56.14,  p=0.0001), with mean inhibition rates of 55.97 ± 0.15%, 54.80 ± 0.12%, and 53.60 ± 0.21% against FS-1, FS-2, and FS-3, respectively (Figure 6B). Despite these differences, SYST2 consistently suppressed mycelial growth by >53% across all three isolates, demonstrating broad and reproducible antagonistic activity against *F. solani* (Figure 6B). Given its high and stable inhibitory efficacy, strain SYST2 was selected for subsequent field biocontrol experiments.

### 3.6. Evaluation of the Field Control Efficacy of B. subtilis SYST2 Against Durian Stem Rot

**Figure 7:**
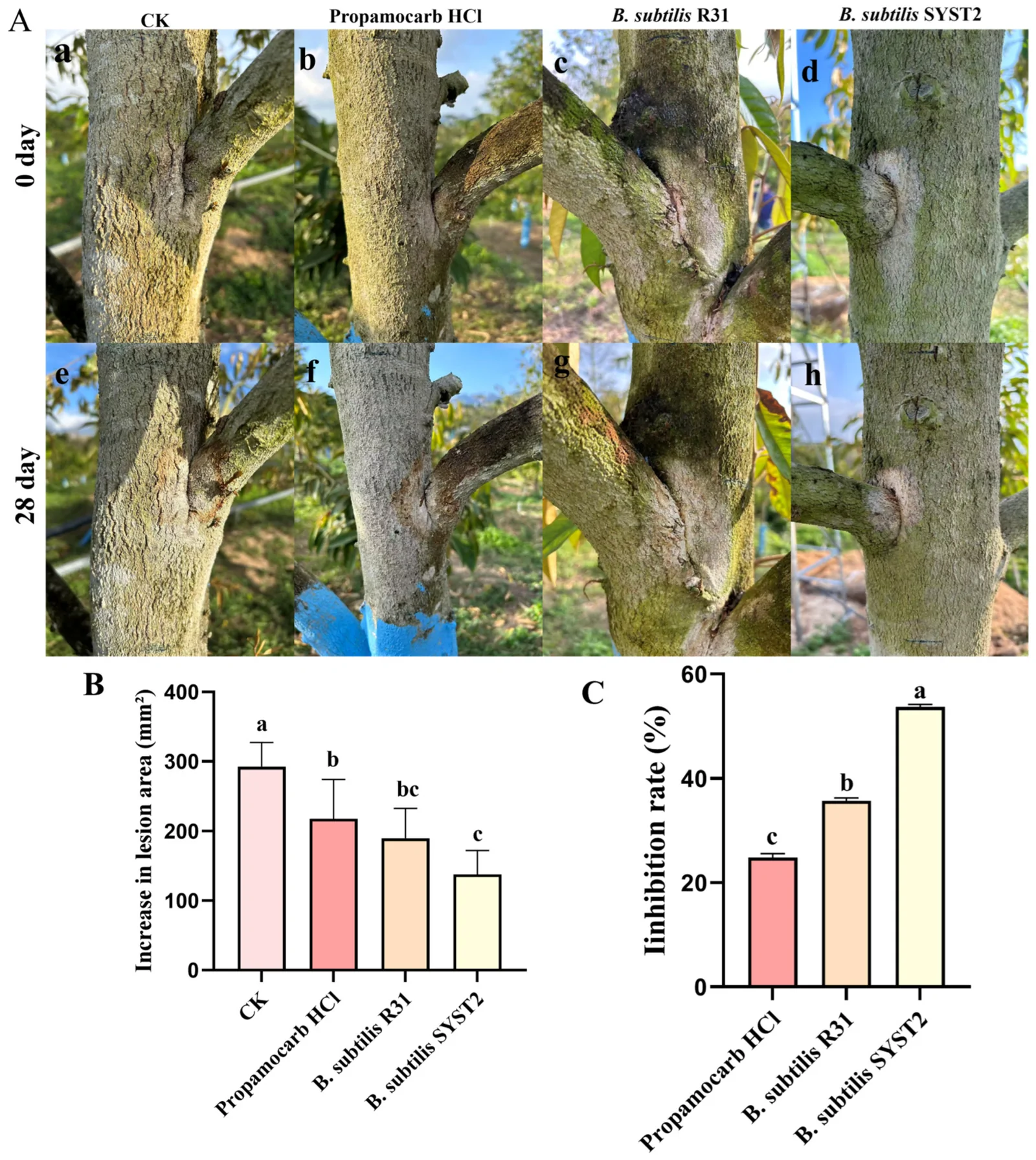
Control efficacy of different treatments against durian stem rot under field conditions. (**A**) Symptoms of durian stem rot in the field: (**a**–**d**) typical lesion symptoms; (**e**–**h**) control effects of different treatments at 28 days after application, e represents the water control, (**f**) represents propamocarb hydrochloride treatment, (**g**) represents *B. subtilis* R31 treatment, and (**h**) represents *B. subtilis* SYST2 treatment. (**B**) Comparison of lesion area increment under different treatments. (**C**) Comparison of disease inhibition rates under different treatments (one-way ANOVA followed by Tukey’s test, *p* < 0.05). Data are presented as mean ± SD. Different lowercase letters indicate significant differences among treatments (one-way ANOVA followed by Tukey’s multiple comparison test, *p* < 0.05).

The field experiment showed that all treatments reduced the expansion of durian stem rot lesions to varying degrees compared to the water-treated control 28 d after application (Figure 7A). Among the tested treatments, there was a highly significant difference among treatments in their inhibitory effects on the expansion of stem rot lesions on durian (F = 10.52, *p* = 0.0007). SYST2 exhibited the smallest increase in lesion area (136.03 ± 15.20 mm^2^), which was significantly lower than that of the water control (292.60 ± 25.40 mm^2^), indicating the highest level of disease suppression. In addition, R31 showed the second-best performance, with a lesion increase of 189.76 ± 18.50 mm^2^, while the effect of propamocarb hydrochloride exhibited relatively weaker efficacy, with a lesion increase of 218.12 ± 22.30 mm^2^ (Figure 7B). Further analysis showed that the inhibition rates of lesion expansion by SYST2, R31, and propamocarb hydrochloride were 53.51%, 35.15%, and 25.46%, respectively (Figure 7C). Overall, these results indicate that SYST2 provided the most effective control of durian stem rot under field conditions, showing greater efficacy than both the tested chemical fungicide and the commercial BCA.

## 4. Discussion

In the present study, *F. solani* was identified as the causal agent of durian stem rot in Sanya, Hainan Province, through pathogen isolation and purification, morphological characterization, molecular identification, and pathogenicity assays. This result is consistent with previous reports from Thailand indicating the involvement of *F. solani* in durian stem rot, suggesting that this pathogen may represent one of the major causal agents of durian stem rot in tropical regions [10]. However, in durian orchards in Thailand, *Lasiodiplodia pseudotheobromae* has been isolated in addition to *Fusarium* spp., whereas most isolates obtained from Hainan samples in the present study belonged to the genus *Fusarium*, and no *L. pseudotheobromae* was isolated. The composition of pathogens associated with durian stem rot may vary among different geographical regions. Whether this disease is caused by mixed infections involving multiple pathogens requires further investigation through expanded sampling and amplicon sequencing approaches.

*F. solani* is widely distributed in tropical and subtropical regions due to its strong environmental adaptability and broad host range [49]. Previous studies have demonstrated that this pathogen can infect various tropical fruits, including dragon fruit, guava, and passion fruit, leading to stem rot, wilt, and root rot diseases, and consequently causing severe economic loss [50,51,52]. Therefore, the findings of this study further enrich the current understanding of durian disease etiology in Hainan Province and provide a theoretical basis for the diagnosis and management of durian diseases in this region.

The survey conducted in this study revealed that the incidence of durian stem rot in the investigated orchards of Sanya reached 26.6%, indicating that the disease has already posed a considerable epidemic risk in local durian plantations. The long-term hot and humid tropical climate conditions in Hainan are conducive to the survival, dissemination, and infection of soil-borne pathogens, thereby facilitating the persistent colonization and spread of *F. solani* in orchard soils and host tissues [53]. Previous studies have demonstrated that high-humidity environments can promote spore germination and mycelial growth of *Fusarium* spp., as well as enhance their soil-borne transmission capacity [54]. In addition, mechanical wounds formed during pruning, grafting, and field management may provide infection courts for pathogen invasion into host tissues, thereby increasing disease incidence [55]. Therefore, orchard management practices that minimize mechanical injuries should be adopted in durian production systems. In addition, BCAs should be applied immediately after pruning, and adequate drainage should be maintained to reduce the risk of disease development.

In recent years, the long-term and extensive use of chemical pesticides has led to increasingly serious issues of pathogen resistance, while also posing potential risks to ecological environments and agricultural product safety [56,57]. Therefore, the development of efficient and environmentally friendly biological control strategies has become an important direction in plant disease management [58]. The results of the present study demonstrated that SYST2 exhibited significant antagonistic activity against *F. solani*. Under field conditions, the control efficacy against durian stem rot reached 53.51%, which was superior to that of the tested chemical fungicides (25.46%) and commercial microbial agents (35.15%), indicating that this strain possesses considerable potential for disease management in tropical orchards. However, compared with previously reported biocontrol efficacies of *Bacillus*-based agents against *Fusarium* diseases, the efficacy level observed in this study remains moderate. Previous studies have demonstrated that the biocontrol performance of *Bacillus* spp. can be significantly enhanced by optimizing application concentration and adding adjuvants, with efficacy reaching over 70% [36]. The moderate efficacy observed in the present study may be attributed to several factors, including the use of a single strain as the BCA, the timing of application relative to disease onset, and the environmental conditions during the trial period. These findings are generally consistent with previous reports. Earlier studies have shown that *B. subtilis* can effectively suppress durian root and stem diseases; previous studies have reported that its biocontrol efficacy against *Fusarium* diseases can reach 57.22%. Lipopeptide compounds produced by *Bacillus* spp. play important roles in inhibiting pathogen growth [36,59]. Compared with chemical pesticides, BCAs exhibit clear advantages in reducing pesticide application, minimizing environmental pollution, and maintaining agricultural ecological balance, which is more consistent with the current demands for sustainable and green agricultural development [60]. Furthermore, the fermentation process of SYST2 is relatively simple, with low production costs, providing economic feasibility for large-scale production and field application. Therefore, biocontrol products based on *B. subtilis* show promising application prospects for the integrated management of durian diseases in Hainan Province.

In this study, SYST2 produced a distinct inhibition zone against *F. solani* in the dual culture assay, indicating that this strain exhibited significant antagonistic activity against the pathogen. However, the types of antagonistic compounds and the specific mechanisms of action of SYST2 (such as antimicrobial metabolites, extracellular enzymes, or VOCs) were not systematically characterized in this study, and the precise mechanisms underlying its inhibitory activity remain unclear. Notably, the SYST2 strain maintained relatively stable disease control efficacy under field conditions, suggesting that this strain possesses strong environmental adaptability and rhizosphere colonization ability. Previous studies have demonstrated that the rhizosphere colonization capacity and environmental adaptability of *B. subtilis* are important factors contributing to its stable biocontrol performance under field conditions [61,62,63]. Future studies integrating metabolomics, genomics, and rhizosphere microbiome analyses could further elucidate the biocontrol mechanisms of SYST2 [64,65] and clarify the molecular basis underlying its biocontrol activity.

Although the present study identified the causal pathogen of durian stem rot and demonstrated the biocontrol potential of the SYST2 strain, several limitations remain. The field evaluation of SYST2 was conducted in a single orchard in Sanya during one growing season, which may not fully represent the diverse environmental conditions across durian-producing regions of Hainan. Therefore, the stability and applicability of SYST2 should be further validated through multi-location and multi-season field trials. Despite these limitations, the present study provides a foundation for the development of SYST2 as a BCA against durian stem rot. These findings also provide a basis for the future commercialization and practical application of SYST2-based biocontrol products.

## 5. Conclusions

In this study, field surveys, morphological characterization, multilocus phylogenetic analyses (*ITS*, *TEF1-α*, and *RPB2*), and pathogenicity assays, including Koch’s postulates, identified *F. solani* as the causal pathogen of durian stem rot in Sanya, Hainan Province, China. To the best of our knowledge, this is the first report of *F. solani* causing stem rot of durian in Hainan Province, China. Among the three isolates, FS-1 exhibited the highest pathogenicity, and the ‘Monthong’ cultivar was more susceptible than ‘Musang King’. In addition, *B. subtilis* SYST2 showed strong antagonistic activity against *F. solani* and effectively reduced disease incidence under field conditions, demonstrating its potential as a BCA. These findings provide a scientific basis for the diagnosis and sustainable management of durian stem rot and support the future development and application of SYST2-based biocontrol products in durian production.

## Data Availability

The fungal ITS sequence data have been deposited in the NCBI GenBank database under accession numbers PZ312000–PZ312002.

## Supplementary Materials

The following supporting information can be downloaded at https://www.mdpi.com/article/10.3390/pathogens15080849/s1. Table S1: PCR primers were used for DNA amplification in this study. Supplementary File S1. DNA sequences of all isolates obtained in this study.
