# Supplementary material for: Isolation, Molecular Characterization, and Biological Control of Fusarium solani Causing Stem Rot of Durian Tree in China
Source: Pathogens. 2026 Aug 14;15(8):849. doi: 10.3390/pathogens15080849 (PMC13516780; doi:10.3390/pathogens15080849)
Supplement: Supplementary file 1 [file pathogens-15-00849-s001.zip › pathogens-4456156-supplementary.pdf]

Supplementary file

Table S1. PCR primers were used for DNA amplification in this study.

Gene/DNA Region	Name	Sequence (5′→3′)	Tm (℃)	Reference
ITS region	ITS1	TCCGTAGGTGAACCTGCGG	56	[41]
	ITS4	TCCTCCGCTTATTGATATGC		
TEF1-α gene	EF1-F	ATGGGTAAGGARGACAAGAC	56	[42]
	EF1-R	ATGGGTAAGGARGACAAGAC		
RPB2	5f2-F	GGGGW GAY CAG AAG AAGGC	56	[43]
	7c-R	CCCAT RGC TTG YTT RCCCAT		

Supplementary File S1. DNA sequences of all isolates obtained in this study.

>FS-1 internal transcribed spacer

CTGAATGTTAGGGGGTTTACGGCGTGGCCGCGCCGCTCTCCAGTTGCGAG
GTGTTAGCTACTACGCAATGGAAGCTGCGGCGGGACCGCCACTGTATTTG
GGGGACGGCGTGTGCCCGCGGGGGGCTCCGCCGATCCCCAACGCCAGGC
CCGGGGGCCTGAGGGTTGTAATGACGCTCGAACAGGCATGCCCGCCAGA
ATACTGGCGGGCGCAATGTGCGTTCAAAGATTCGATGATTCACTGAATTCT
GCAATTCACATTACTTATCGCATTTCGCTGCGTTCTTCATCGATGCCAGAGC
CAAGAGATCCGTTGTTGAAAGTTTTAATTTATTTGCTTGTTTTACTCAGAAG
AAACATTATAGAAACAGAGTTAGGGGGTCCTCTGGCGGGGGCGGCCCGT
GTTACGGGGCCGTCTGTTCCCGCCGAAGCAACGTTTAGGTATGTTCACAG
GGTTGATGAGTTGTATAACTCGGTAATGATCCCTCCGCAGGTAACC

>FS-2 internal transcribed spacer

CTGGCCTGATTCGAGGTCACTTCAGAAGTTGGGTGTTTTACGGCATGGCCG
CGCCGCTCTCCAGTTGCGAGGTGTTAGCTACTACGCAATGGAAGCTGCGG

CGGGACCGCCACTGTATTTGGGGGACGGCGCGTGCCCACAGGGGGCGCC
TCGCCGATCCCCAACGCCAGGCCCGGGGGCCTGAGGGTTGTAATGACGCT
CGAACAGGCATGCCCGCCAGAATACTGGCGGGCGCAATGTGCGTTCAAA
GATTCGATGATTCACTGAATTCTGCAATTCACATTACTTATCGCATTTCGCT
GCGTTCTTCATCGATGCCAGAGCCAAGAGATCCGTTGTTGAAAGTTTTAAT
TTATTTGCTTGTTTACTCAGAAGATACATATAGCAACAGAGTTAGGGGTCC
TCTGGCGGGGGCGGCCCGTTTTACGGGGCCGTCTGTTCCCGCCGAAGCAA
CGTTTTAGGTATGTTCACAGGGTTGATGAGTTGTTTAGACTCGGTAATGATC
CCTCCGCAGTTACC

>FS-3 internal transcribed spacer

ATCGAGTCAGCGTCTCACCCTGTGAACTACCTAAAACGTTGCTTCGGCGG
GAACAGACGGCCCCGTAAAACGGGCCGCCCCCGCCAGAGGACCCCTAA
CTCTGTTGCTATATGTATCTTCTGAGTAAACAAGCAAATAAATTAAAACTTT
CAACAACGGATCTCTTGGCTCTGGCATCGATGAAGAACGCAGCGAAATG
CGATAAGTAATGTGAATTGCAGAATTCAGTGAATCATCGAATCTTTGAACG
CACATTGCGCCCGCCAGTATTCTGGCGGGCATGCCTGTTCGAGCGTCATTA
CAACCCTCAGGCCCCCGGGCCTGGCGTTGGGGATCGGCGAGGCGCCCCC
TGTGGGCACGCGCCGTCCCCCAAATACAGTGGCGGTCCCGCCGCAGCTTC
CATTGCGTAGTAGCTAACACCTCGCAACTGGAGAGCGGCGCGGCCATGCC
GTAAAACACCCAACTTCTGAATGTTGACCTCGAATCAGGTAGGAATACCC
GCTGAACTTAAGCATATCAAAAA

>FS-1 translation elongation factor 1-alpha

TGTATCGGTAGTCGACTCTGGCAGTCGACCACCGTAAGTCAAACCCTCAT
CGCGATCTGCTTATCTCGGGTCGTGGAACCCCGCCTGGTATCTCGGGCGGG
GTATTCATCAGTCACTTCATGCTGACAATCATCTACAGACCGGTCACTTGA
TCTACCAGTGCGGTGGTATCGACAAGCGAACCATCGAGAAGTTCGAGAA
GGTTGGTGACATCTCCCCCGATCGCGCCTTGATATCACACATCGAATCCCC
TCCCTCACGATCCGCGCTGCGCCCGCTTCTCCCGAGTCCCAAAATTTTTGC
GGTGCGACCGTGAATTTTTTTGGTGGGGCATTTTACCCCGCCACTCGGGCG
ACGTTGGACAAAGCCCTGATCCCTGCACACAAAAAACACCAAACCCTCT
TGGCGCGCATCATCACGTGGTTGACCACAGACGCTAACCGACTCAACAA
CAGGAAGCCGCTGAGCTCGGTAAGGGTTCCTTCAAGTACGCCTGGGTCCT

TGACAAGCTCAAGGCCGAGCGTGAGCGTGGTATCACCATCGACATTGCCC
TCTGGAAGTTCGAGACTCCCCGCTACTATGTCACCGTCATTGGTATGTCGC
TGCCGTTTCTCTCAATCATGTCTCACCACTAACAATCAACAGACGCCCCCG
GCCACCGTG

>FS-2 translation elongation factor 1-alpha

AAGGTCTCGGCCTCGACTCTGGCAGTCGACCACCGTAAGTCAAACCCTCA
TCGCGATCTGCTTATCTCGGGTCGTGGAACCCCGCCTGGCATCTCGGGCGG
GGTATTCATCAGTCACTTCATGCTGACAATCATCTACAGACCGGTCACTTG
ATCTACCAGTGCGGTGGTATCGACAAGCGAACCATCGAGAAGTTCGAGA
AGGTTGGTGATATCTCCCCCGATCGCGCCTTGCTATTCCACATCGAATTCC
CTCCCTCGCGATACGCTCTGCGCCCGCTTCTCCCGAGTCCCAAAATTTTTG
CGGTCCAACCGCAATTTTTTTGGTGGGGCATTTACCCCGCCACTCGGGCG
ACGTTGGACAAAGCCCTGATCCCTGCACACAAAAAACACCAAACCCTCT
TGGCGCGCATCATCACGTGGTTCACACCAGACGCTAACCGGTTTAACAAT
AGGAAGCCGCTGAGCTCGGTAAGGGTTCCTTCAAGTACGCCTGGGTCCTT
GACAAGCTCAAGGCCGAGCGTGAGCGTGGTATCACCATCGACATTGCCCT
CTGGAAGTTCGAGACTCCCCGCTACTATGTCACCGTCATTGGTATGTTGCT
GTCACCTGTCTCACGCATGTCTCACCACTAACAATCAACAGACGCCCCCG
GCCACCGTG

>FS-3 translation elongation factor 1-alpha

CTAGTATCGTCCGTCGACTCTGGCAAGTCGACCACCGTAAGTCAAACCCT
CATCGCGATCTGCTTATCTCGGGTCGTGGAACCCCGCCTGGCATCTCGGGC
GGGGTATTCATCAGTCACTTCATGCTGACAATCATCTACAGACCGGTCACT
TGATCTACCAGTGCGGTGGTATCGACAAGCGAACCATCGAGAAGTTCGAG
AAGGTTGGTGATATCTCCCCCGATCGCGCCTTGCTATTCCACATCGAATTC
CCTCCCTCGCGATACGCTCTGCGCCCGCTTCTCCCGAGTCCCAAAATTTTT
GCGGTCCAACCGCAATTTTTTTGGTGGGGCATTTACCCCGCCACTCGGGC
GACGTTGGACAAAGCCCTGATCCCTGCACACAAAAAACACCAAACCCTC
TTGGCGCGCATCATCACGTGGTTCACACCAGACGCTAACCGGTTTAACAA
TAGGAAGCCGCTGAGCTCGGTAAGGGTTCCTTCAAGTACGCCTGGGTCCT
TGACAAGCTCAAGGCCGAGCGTGAGCGTGGTATCACCATCGACATTGCCC
TCTGGAAGTTCGAGACTCCCCGCTACTATGTCACCGTCATTGGTATGTTGC

TGTCACCTGTCTCACGCATGTCTCACCACTAACAATCAACAGACGCCCCC
GGCCACCGTG

>FS-1 RNA polymerase II second largest (*RPB2*) gene

GGGGAGACCAGAAGAAGGCCGCAAGCTCCACTGCTGGTGTCTCGCAGGT
GTTGAACAGATACACCTTTGCATCTACCCTTTCGCATTTGCGGCGAACCAA
CACCCCTATTGGACGAGATGGAAAGCTCGCCAAGCCCCGTCAGCTACAC
AACACTCATTGGGGTCTGGTCTGTCCAGCCGAGACGCCTGAGGGTCAGGC
TTGTGGTCTGGTCAAGAACTTGTCACTGATGTGCTACGTCAGTGTCGGCTC
TCCCTCCGAACCCCTGATCGAGTTCATGATCAACCGAGGTATGGAAGTCG
TGGAAGAGTACGAACCCCTGAGATACCCCCATGCCACCAAGATTTTCGTC
AACGGTGTCTGGTGTGGTGTTCACTCAGACCCCAAGCATCTCGTCAGCCA
GGTCCTGGACACACGACGAAAGTCGTACCTGCAGTACGAGGTGTCACTTG
TTCGTGACATTCGAGATCGAGAGTTCCAGGTCTTCTCCGACGCTGGCCGA
GTCATGAGGCCGGTCTTTACGGTTCAGCAGGAGGATGACCACGAGTCTGG
TATTGCCAAGGGAGCTCTGGTTCTGACCAAGGACCTTGTCAACAAGCTTG
CCAAGGAGCAAGCGGAGCCACCAGAGGACCCATCACAGAAGATTGGAT
GGGAGGGTCTGATCCGAGCCGGAACCATCGAGTACCTCGATGCTGAGGA
AGAGGAGACGGCTATGATTTGCATGACTCCTGAGGATCTTGACCTCTACC
GCATGCAAAAGGCTGGTTACGTCGTGGATGACGATAACACGGACGACCC
GAACAGGAGATTGAAGACCAAGACGAACCCCACAACTCACATGTACACT
CATTGTGAGATTCACCCCAGTATGATTCTCGGCATTTGCGCCAGTATCATTC
CTTTCCCAGATCACAACCAGGTATGTGCTCTTGTTTTACAGTGGTGCCAGC
ACAGCTAACAGTATGTAGTCGCCTCGTAACACCTACCAATCTGCCATGGG
CAAGCAAGCCATGGG

>FS-2 RNA polymerase II second largest (*RPB2*) gene

GGGGAGACCAGAAGAAGGCTGCAAGCTCTACTGCTGGTGTCTCTCAGGT
GTTGAACAGATACACCTTTGCATCCACTCTTTCGCATTTGCGACGAACCAA
CACCCCTATCGGACGAGATGGAAAGCTCGCCAAGCCCCGCCAGCTACAC
AACACCCATTGGGGTCTGGTCTGTCCAGCCGAGACGCCTGAGGGTCAGG
CTTGTGGTCTGGTCAAGAACTTGTCCCTGATGTGCTACGTCAGTGTCGGCT
CTCCCTCCGAACCTCTGATTGAGTTCATGATCAACCGAGGTATGGAAGTC
GTGGAAGAGTACGAGCCTCTGAGATACCCGCATGCTACCAAGATCTTTGT

CAATGGTGTCTGGTGTGGTGTTCACTCAGACCCCAAGCATCTCGTCAGCC
AGGTCCTGGACACACGACGAAAGTCATACCTGCAGTATGAGGTGTCACTT
GTTCGTGACATTCGAGATCGAGAGTTCAAGGTCTTCTCCGACGCTGGCCG
AGTCATGAGGCCGGTCTTTACGGTTCAGCAGGAGGATGACCACGAGTCTG
GTATTGCCAAGGGAGCTTTGGTTCTGACCAAGGACCTTGTCAACAAGCTT
GCTAAGGAGCAGGCGGAGCCACCAGAGGACCCATCAATGAAGATTGGAT
GGGAGGGTCTGATCCGAGCCGGAACCATTGAGTACCTCGATGCCGAGGA
AGAGGAGACGGCTATGATTTGCATGACTCCTGAGGATCTTGATCTCTATCG
CATGCAAAAGGCTGGTTACGTCGTAGATGACGATAACACGGACGACCCC
AACAGGAGATTGAAGACCAAGACGAACCCCACAACTCACATGTACACTC
ATTGTGAGATTCACCCCAGTATGATTCTTGGAATTTGTGCCAGTATCATTCC
CTTCCCCGATCACAACCAGGTATGTGCCCATGATTCAACGTGATGCTAACG
CGGCTAACAATATGTAGTCACCCCGTAACACCTACCAATCTGCCATGGGC
AAGCAAGCCATGGG

>FS-3 RNA polymerase II second largest (*RPB2*) gene

GGGGAGACCAGAAGAAGGCTGCAAGCTCTACTGCTGGTGTCTCTCAGGT
GTTGAACAGATACACATTTGCATCCACTCTTTCGCATTTGCGACGAACCAA
CACCCCCATTGGACGAGATGGAAAGCTCGCCAAGCCCCGTCAGCTACAC
AACACCCATTGGGGTCTGGTCTGTCCAGCCGAGACACCCGAGGGCCAGG
CTTGTGGTCTGGTCAAGAACTTGTCTCTGATGTGTTACGTCAGTGTCGGCT
CTCCTTCTGAACCCCTGATCGAGTTCATGATCAACCGAGGTATGGAAGTC
GTGGAAGAGTACGAGCCCCTGAGGTACCCTCATGCTACCAAGATCTTTGT
CAATGGTGTTTGGTGCGGTGTCCACTCGGACCCAAAGCATCTCGTCAGCC
AGGTCCTGGACACACGACGAAAGTCGTACCTGCAGTATGAGGTGTCGCTT
GTTCGTGACATTCGAGATCGAGAGTTCAAGGTCTTCTCCGACGCTGGCCG
AGTCATGAGGCCGGTCTTTACGGTCCAGCAGGAGGATGATCACGAGTCTG
GTATCGCCAAGGGAGCTTTGGTTTTGACCAAGGACCTTGTCAACAAGCTT
GCGAAGGAGCAGGCGGAGCCACCAGAGGACCCATCACAGAAGATTGGA
TGGGAGGGTCTGATCCGAGCCGGAACCATCGAGTACCTCGATGCTGAGG
AAGAAGAGACGGCTATGATTTGCATGACCCCTGAGGATCTTGATCTCTATC
GTATGCAAAAGGCTGGTTACGTCGTAGATGACGATAACACGGACGACCCC
AACAGGAGATTGAAGACCAAGACGAACCCCACAACTCACATGTACACTC
ATTGTGAGATTCACCCCAGTATGATTCTTGGCATTTGTGCCAGTATCATTCC

CTTTCCCGATCACAACCAGGTATGTGCCCTTCATTCAACGTGATGCTAGCG
CAGCTAACAATATGTAGTCACCTCGTAACACCTACCAATCTGCCATGGGC
AAGCAAGCCATGGG
